# Low bone mineral density is related to atherosclerosis in postmenopausal Moroccan women

**DOI:** 10.1186/1471-2458-9-388

**Published:** 2009-10-14

**Authors:** Ihsane Hmamouchi, Fadoua Allali, Hamza Khazzani, Loubna Bennani, Leila EL Mansouri, Linda Ichchou, Mohammed Cherkaoui, Redouane Abouqal, Najia Hajjaj-Hassouni

**Affiliations:** 1 Laboratory of Information and Research on Bone Diseases (LIRPOS). Department of Rheumatology, El Ayachi hospital, University Hospital of Rabat-Sale, Morocco; 2 Laboratory of Biostatistical, Clinical and Epidemiological Research (LBRCE). Faculty of Medicine and Pharmacy, Rabat, Morocco; 3 Department of Radiology, Cheikh Zayd University Hospital, Rabat, Morocco

## Abstract

**Background:**

Some studies have implicated several possible metabolic linkages between osteoporosis and vascular calcification, including estrogen deficiency, vitamin D excess, vitamin K deficiency and lipid oxidation products. Nevertheless, it remains unclear whether osteoporosis and atherosclerosis are related to each other or are independent processes, both related to aging. The aim of this cross-sectional study was to evaluate the correlation between arterial thickening and bone status in a sample of apparently healthy Moroccan women.

**Methods:**

Seventy-two postmenopausal women were studied. All patients were without secondary causes that might affect bone density. Bone status was assessed by bone mineral density (BMD) in lumbar spine and all femoral sites. Arterial wall thickening was assessed by intima-media thickness (IMT) in carotid artery (CA) and femoral artery (FA). Prevalent plaques were categorized into four groups ranging from low echogenicity to high echogenicity.

**Results:**

The mean age was 59.2 ± 8.3 years. 84.7% had at least one plaque. By Spearman Rank correlation, CA IMT was negatively correlated to Femoral total BMD (r = -0.33), Femoral neck BMD (r = -0.23), Ward triangle BMD (r = -0.30) and Trochanter BMD (r = -0.28) while there was no association with lumbar BMD. In multiple regression analysis, CA IMT emerged as an independent factor significantly associated with all femoral sites BMD after adjusting of confounding factors. FA IMT failed to be significantly associated with both Femoral and Lumbar BMD. No significant differences between echogenic, predominantly echogenic, predominantly echolucent and echolucent plaques groups were found concerning lumbar BMD and all femoral sites BMD

**Conclusion:**

Our results demonstrate a negative correlation between bone mineral density (BMD) qnd carotid intima-media thickness (IMT) in postmenopausal women, independently of confounding factors. We suggest that bone status should be evaluated in patients with vascular disease to assess whether preventive or therapeutic intervention is necessarry.

## Background

Atherosclerosis and osteoporosis are two of the most common diseases that are correlated with the health of elderly women. Both are often seen in the same individual. These conditions progress silently until a fracture or myocardial infarction occurs [1,2].

Previous studies have shown an association between osteoporosis and aortic [3-5] and carotid atherosclerosis [6], cardiovascular mortality [7,8], stroke [7] and all-cause mortality [9] in women and men. Both osteoporosis and vascular calcification have largely been attributed to the aging process. Recent studies have shown, however, that arterial calcification is a highly regulated process, with intriguing similarities to bone turnover [10,11] that may be age-independent. The nature of the relationship between osteoporosis and atherosclerosis remains unclear. Some studies have implicated several metabolic linkages between osteoporosis and vascular calcification. These include estrogen deficiency [12], vitamin D excess [13], vitamin K deficiency [14], and lipid oxidation products [15].

Measurement of the far-wall intima-media thickness (IMT) of the common carotid artery (CA) and femoral artery (FA) by high-resolution ultrasonography has been established as a clinically useful index for identifying early-stage general and local atherosclerosis in lower extremities, because CA IMT is correlated strongly with the presence of coronary artery disease [16-18] and FA IMT with local atherosclerosis [19].

The aim of this cross-sectional study was to evaluate the correlation between bone mineral density and artery IMT, a measure of preclinical atherosclerosis, in a sample of apparently healthy postmenopausal Moroccan women.

## Methods

### Subjects

The study involved 72 consecutive, ambulatory, Moroccan, postmenopausal women living in urban centre of Morocco who were sent to our outpatient Bone Densitometry Center. Recruitment was based on voluntary enrolment. All subjects were referred to this center for osteoporosis risk factors, including menopause. Osteoporosis was assessed by BMD and defined according to the world Health Organization (WHO) (<2,5 standard deviation of normal values for young people). Study inclusion criteria were: (1) postmenopausal status (at least 1 year of menopause). Exclusion criteria included having a history of: (1) taking drugs known to influence bone metabolism in the past 2 years, such as vitamin D, calcium, corticosteroids, bisphosphonates and hormone replacement therapy; (2) musculoskeletal, thyroid, parathyroid, adrenal, hepatic, or renal disease; (3) malignancy,(4) hysterectomy; and (5) history of atherosclerotic heart disease or stroke. The study was approved by ethical committee of University-hospital Mohamed V Souissi and all participants provided written consent.

### Data collection and measurements

Each patient completed a questionnaire on sociodemographic parameters, atherosclerosis and osteoporosis risk factors. The age of menopause, the time since menopause, the personal history of cigarette smoking or alcohol intake and the number of pregnancies were recorded.

For the evaluation of physical activity, we used the short form of the International Physical Activity Questionnaire (IPAQ) [20]. The items of IPAQ were structured to provide separate scores on walking, moderate-intensity and vigorous-intensity activity. Computation of the total score requires summation of the duration (in minutes) and frequency (days) of walking, moderate-intensity and vigorous-intensity activities [21].

### Anthropometric data

Weight and height were measured without clothes or shoes at the time of bone densitometry measurements. The Body mass index (BMI) was calculated as body weight/height^2 ^(kg/m^2^). The inter-assay CV of QC pools was 3%.

### Biochemical measurements

Morning fasting samples of venous blood were taken. Serum total cholesterol (CT, enzymatic colorimetric methods; reference value (RV) 1.4-2.4 g/l), serum high-density lipoprotein (HDL, enzymatic colorimetric methods; RV,0.50-0.75 g/l), low-density lipoprotein (LDL, enzymatic colorimetric methods; RV 0.65-1.75 g/l), triglycerides (TG, enzymatic colorimetric methods; RV 0.22-2.00 g/l) were measured. Serum Calcium (Ca, kinetic colorimetric assay, RV, 84-97 mg/l); Phosphorus (P, kinetic colorimetric assay, RV, 27-45 mg/l) and serum creatinine(Cr, kinetic colorimetric assay, RV,5.1-9.5 mg/l) were also measured. Osteocalcin (OC; ECLIA (electrochimiluminescence); RV, 15-46 ng/ml; Elecsys N-Mid Osteocalcin), fasting urine cross-linked carboxy-terminal telopeptide of type I collagen corrected by urinary creatinine (CTX, ECLIA (electrochimiluminescence); RV, 1.008 ng/ml; Elecsys bCross laps) were determined as bone turnover markers. All theses measurements have been made using Intégra 400 (Roche diagnostics; Mannheim, Germany). Moreover, Serum 25 (OH)VitD (25-hydroxyvitamin D; chimiluminescence; (RV) 20-60 μg/ml; Liaison, Diasorin) and intact PTH (PTH; ECLIA (electrochimiluminescence); RV, 15-65 pg/ml; Elecsys Intact PTH) were assayed.

### Bone mineral density (BMD)

Lumbar spine, trochanter, femoral neck and total hip BMD were measured by dual-energy X-ray absorptiometry with a Lunar prodigy densitometer. Daily quality control was carried out by measurement of a Lunar phantom. At the time of the study, phantom measurements showed stable results. The phantom precision expressed as the CV(%) was 0.08. Both T and Z scores were obtained. In the T-score calculations, the manufacturer's ranges for European population reference were used because of the absence of a Moroccan data base.

### Ultrasonography of the carotid artery (CA) and femoral artery (FA)

Ultrasonographic examination of the CA and FA was performed in the supine position by the use of high-resolution B-mode ultrasonography performed with an ultrasonography scanner (Xp10 128 ART-upgraded; Acuson, Mountain View, CA), as previously described [22]. To avoid inter-observer variability, all measurements were performed by the same examiner who was unaware of subject characteristics. We recorded atherosclerotic plaques from six sites of the carotid artery: The near and far walls of both internal carotid arteries, the bifurcation segment of the common carotid artery, and the common carotid artery from the bifurcation segment to as far downstream of the supraclavicular region as technically possible; if more than one plaque was present at one of the six sites, the thickest plaque was chosen for analysis. Moreover ultrasound (US) imaging of the femoral arteries was carried out at the level of the bifurcation on both the right and left sides. Atherosclerotic plaque was defined as localized protrusion of the internal part of the vessel wall into the lumen. Plaque morphology in terms of echogenicity, defined as reflectance of the emitted ultrasound signal, was assessed by the use of a visual analogue technique along a gray scale [22]. Plaques that appeared black or almost black (like fluent blood inside the vessel) were described as grade 1 (echolucent, low echogenic), whereas plaques that appeared white or close to white (echogenic), similar to the bright echo zone produced by the media-adventitia interface in the far wall of the carotid artery, were classified as grade 4. Grade 2 and grade 3 plaques were interpolated between grades 1 and 4 along the black-white scale, the grade 2 plaques consisting of more echolucent than echogenic materials and the grade 3 plaques vice versa. If one single plaque was present and the echogenicity inside this plaque was heterogeneous, the dominant echogenicity determined the grading. When more than one plaque was present, the echogenicity was graded considering the overall plaque area [22]. The mean coefficient of variation [23] for the difference between IMT measurement obtained in repeated examinations performed by the same examiner was 3.2%.

### Statistical analysis

Statistical analysis was performed with the Windows 13.0 version of SPSS software (SPSS Inc., Chicago, IL, USA). Values are expressed mean ± S.D or percentages. Normality of the data was tested with a one-sample Kolmogorov Smirnov test to indicate the appropriateness of parametric testing. The correlation coefficients were calculated by Pearson Rank correlation analyses due to normal distribution of various clinical variables. Multiple regression analysis was performed to assess independent association with lumbar spine BMD and femoral BMD. For the relation between plaque echogenicity and BMD, the comparison between groups was made using analysis of covariance (ANOVA). P-values of < 0.05 were considered as statistically significant.

## Results

### Clinical variables, intima-media thickness and bone density of patients

Characteristics of participants enrolled in this cross sectional study are shown in table 1. The mean age and BMI was 59.2 ± 8.3 years and 27.7 ± 4.5 kg/m^2 ^respectively. Of the 72 persons included in the study, 55.6% were osteoporotic and 84.7% had at least one plaque. The means of triglycerides, serum total Cholesterol, high-density lipoprotein and low-density lipoprotein were within normal limits for reference laboratory. The means of CA and FA IMT were to 0.8 ± 0.4 mm and 0.8 ± 0.3 mm respectively. 36.1% of Patients had femoral plaque and 59.7% had carotid artery plaque. Plaques were distributed, according to the echogenity, as follows: 31.9% Type I echolucent, 16.7% Type II predominantly echolucent, 9.7% type III predominantly echogenic and 26.4% type IV echogenic.

**Table 1:** Characteristics of study participants

**Number**	**72** ***Mean (SD)***
Age (years)	59.2 ± 8.3
Years since menopause (years)	12.0 ± 8.2
Number of pregnancies	5.2 ± 3.4
Body mass index (kg/m2)	27.7 ± 4.5
Physical activity score (min/week)	3448 ± 1053
Systolic blood pressure (mmHg)	137 ± 17
Serum level	
Triglyceride (g/l)	1.3 ± 0.7
Total Cholesterol (g/l)	2.1 ± 0.3
high-density lipoprotein (g/l)	0.5 ± 0.1
low-density lipoprotein (g/l)	1.2 ± 0.3
CA IMT (mm)	0.8 ± 0.4
FA IMT (mm)	0.8 ± 0.3
Lumbar spine BMD (g/cm^2^)	0.917 ± 0.172
Trochanter BMD (g/cm^2^)	0.669 ± 0.121
Femoral neck BMD (g/cm^2^)	0.823 ± 0.109
Ward triangle BMD (g/cm^2^)	0.645 ± 0.140
Femoral total BMD (g/cm^2^)	0.860 ± 0.111
	*Number (Percentage)*
Current smoking	2 (2.8)
Osteoporosis	40 (55.6)
History of personal peripheral osteoporotic fractures	13 (18.1)
Number of individuals with plaque	61 (84.7)
Femoral Plaque	26 (36.1)
Carotid Plaque	43 (59.7)
Plaques echogenicity	
No plaque	11 (15.3)
Type I echolucent	23 (31.9)
Type II predominantly echolucent	12 (16.7)
Type III predominantly echogenic	7 (9.7)
Type IV echogenic	19 (26.4)

CA: carotid artery, IMT: intima-media thickness, FA: Femoral artery

The means of serum Calcium, Phosphorus, 25OH D3, intact PTH, CTX and osteocalcin were within normal limits for reference laboratory (results not shown in table 1).

There were no significant differences among the groups osteoporotic versus no osteoporotic in age, age of menopause, BMI, Physical activity score. Serum Calcium, Phosphorus, 25 OH D3, total Cholesterol, high-density lipoprotein, low-density lipoprotein, Triglycerides, Intact PTH, Osteocalcine, Cross laps and Creatinine were not different and except for 25OH D3, were within normal limits for reference laboratory. On the basis of vascular ultrasound examination, the prevalence of artery plaques for any of the sites examined was not significantly different in patients with or without osteoporosis (p = 0.393). No significant association was observed concerning the plaque morphologic echography (p = 0.241). Results were shown table 2.

**Table 2:** Characteristics of study participants after splitting them in osteoporotic and no osteoporotic

	**Osteoporosis**	**Non osteopotic patients**	**P value^a^**
Number	40	32	
			
Number of individuals with plaque			
Femoral Plaque	19 (47.5)	16 (50.0)	0.393
Carotid Plaque	2 (5.0)	1 (3.1)	1.000
			
Plaques echogenicity			
Type I echolucent	5 (12.5)	6 (22.2)	0.241
Type II predominantly echolucent	8 (20.0)	2 (06.2)	
Type III predominantly echogenic	3 (7.5)	7 (21.8)	
Type IV echogenic	3 (7.5)	2 (06.2)	
			
*Mean (SD)*			
Age (years)	59.1 ± 6.2	58.7 ± 6.5	0.797
Menopause age (yeras)	48.6 ± 5.5	48.0 ± 6.2	0.724
Number of pregnencies	6.0 ± 2.6	4.0 ± 2.0	0.193
Body mass index (kg/m2)	28.3 ± 4.0	26.3 ± 5.1	0.193
Physical activity score	2160 ± 1546	3690 ± 2134	0.200
			
Serum level			
Triglyceride (g/l)	1.25 ± 0.41	0.97 ± 0.32	0.094
Total Cholesterol (g/l)	2.05 ± 0.30	2.06 ± 0.31	0.934
high-density lipoprotein (g/l)	0.55 ± 0.12	0.59 ± 0.19	0.572
low-density lipoprotein (g/l)	1.25 ± 0.24	1.27 ± 0.24	0.645
Calcium (mg/l)	97.33 ± 5.87	98.14 ± 5.15	0.533
Phosphorus (mg/l)	35.80 ± 4.60	37.20 ± 3.20	0.348
25 OH D3 (μg/l)	15.36 ± 7.46	16.01 ± 5.77	0.699

### Correlations between CA IMT, FA IMT and clinical variables and BMD

**Figure 1:**
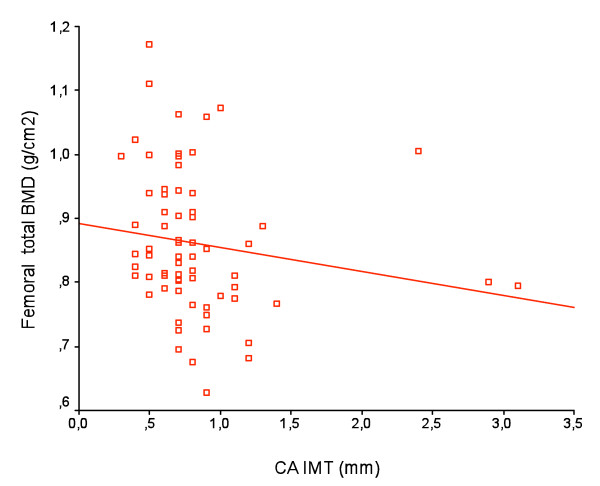
**Correlation of Carotid artery intima-media thickness (CA IMT) with femoral total BMD**. A significant negative correlation was found between Femoral total BMD and CA IMT (r = -0.237, p = 0.006) by Pearson analysis.

Table 3 shows the summary of correlations of IMT with clinical variables including BMD by Pearson Rank correlation. Only Age was positively correlated with both CA and FA IMT. CA IMT was negatively correlated to Trochanter BMD, Femoral neck BMD, Ward triangle BMD and Femoral total BMD (figure 1) while there was no association with lumbar BMD. FA IMT failed to be significantly associated with both Femoral and Lumbar BMD.

**Table 3:** Correlations of IMT in each segment with clinical characteristics and BMD

**Clinical variables**	**CA IMT**	**FA IMT**
Age (years)	0.227 *	0.309 *
Menopause age (years)	-0.081	0.005
Body mass index (kg/m2)	0.087	0.042
Systolic blood pressure (mmHg)	0.038	-0.195
Triglyceride (g/l)	-0.077	-0.015
High-density lipoprotein (g/l)	0.053	0.235
Low-density lipoprotein (g/l)	0.003	0.275
Physical activity score	0.024	-0.094
Lumbar spine BMD (g/cm^2^)	-0.131	0.071
Femoral total BMD (g/cm^2^)	-0.330 *	-0.054
Femoral neck BMD (g/cm^2^)	-0.237 *	0.041
Ward triangle BMD (g/cm^2^)	-0.301 *	-0.078
Trochanter BMD (g/cm^2^)	-0.280 *	-0.110

Values indicate bivariate correlation coefficients (Pearson's rho) obtained from 72 healthy Moroccan women. CA: carotid artery, IMT: intima-media thickness, FA: Femoral artery.

* p < 0.05

### Multiple regression analysis of factors independently associated with BMD

In multiple regression analysis, CA IMT was an independent factor significantly associated with Trochanter BMD, Ward triangle BMD, Femoral Neck BMD, femoral total BMD but not lumbar spine BMD after adjusting of age, age of menopause, serum LDL cholesterol, systolic blood pressure and physical activity. Serum LDL cholesterol emerged as an independent factor significantly associated with femoral BMD (table 4).

**Table 4:** Stepwise regression analysis of factors independently associated with BMD included CA IMT.

	**FT BMD**	**W BMD**	**FN BMD**	**T BMD**
	
	**β**	**β**	**β**	**β**
Age	-0.015*	-0.019*	-0.001*	-0.014*
LDL	0.238*	0.151	0.241*	0.078
CA IMT	-0.467*	-0.054*	-0.383*	-0.489*

LDL: low-density lipoprotein, CA: carotid artery, IMT: intima-media thickness, BMD: bone mineral density, FT: Femoral total, W: Ward triangle, FN: Femoral neck, T: Trochanter.

Confounding factors are: Age, Menopause Age, systolic blood pressure, physical activity, LDL and CA IMT

Values are standard regression coefficient (β). *p < 0.05

### BMD and plaques echogenicity

No significant differences between echogenic, predominantly echogenic, predominantly echolucent and echolucent plaques groups were found concerning lumbar BMD and all femoral sites BMD (data not shown).

## Discussion

The most important finding in the present study is that CA IMT emerged as an independent factor that is associated significantly with Trochanter BMD, Femoral neck BMD, Ward triangle BMD and Femoral total BMD, but not with lumbar spine BMD. The association between trabecular or cortical osteoporosis and calcification in different vascular beds may vary, and should be considered when comparing the reported findings. In many studies evaluating the association between low bone mass and vascular calcification, there was an independent association between BMD at cortical sites, as represented by low hip BMD and vascular calcification [6,12,24]. However, studies measuring BMD at predominantly trabecular sites, namely spinal sites, failed to demonstrate this association [4,6,24,25]. In the past, this lack of association had been attributed to methodological pitfalls related to Dual Energy X-ray Absorptiometry (DXA) measurements. In fact, DXA measurements of the spine are confounded by the presence of calcified plaques in adjacent vessels and osteophytes [26]. Nevertheless, the lack of an association at trabecular sites was confirmed by the Sinnot study [27] that used highly sensitive and specific quantitative computerized tomography (QCT) measures of exclusively trabecular bone of the vertebrae. By contrast, previous studies have shown a negative association between lumbar BMD and IMT, but this lack of association has also been described in hemodialysis patients [28].

Our results indicate that FA IMT is not associated significantly with BMD. Our data seem to be in contrast to the earlier findings [5-8] that found a significant association between FA IMT and bone status. It is also conceivable that atherosclerotic distribution within the vascular tree may vary considerably between vascular beds, and this may explain some of the apparently conflicting findings in the literature [24,29,30].

Another finding in the present study was that CA IMT is associated significantly with all femoral site BMD independently of age. Indeed, both osteoporosis and atherosclerosis increase with age, but the relationship between them has remained unclear [31,32]. In some studies, the apparent association between BMD and vascular calcification disappeared after adjusting for age [25,31-33], while in others, the association was independent of age [3,14,33].

The biological explanation for the possible relationship between atherosclerosis and osteoporosis is unclear at present, but several hypotheses have been proposed. Oxidized lipids [15], impaired vitamin K status [14], homocysteine, and high levels of osteoprotegerin [10,11] are among the factors that may contribute to the association between atherosclerosis and osteoporosis. Hamerman [34] reviewed several possibilities, and pointed out that inflammation is likely to be one of the processes that influence atherogenesis and bone loss. Many of the inflammatory mediators driving atherogenesis in the arterial wall are known to be in the circulation as markers of cardiovascular risk. These could gain access to bone, where, together with local cytokines, they could enhance the release of osteoblastic factors, that in turn promote osteoclastogenesis.

Several researchers have suggested a role for hyperlipidemia and lipid oxidation [15] and inflammation [34]. *In vitro *studies have shown that oxidized lipids promote osteoblastic differentiation of vascular cells and inhibit such differentiation in bone cells [35]. One possible mechanism by which this may occur is through accumulation of oxidized lipids in tissue so as to mimic chronic infection, thereby stimulating an immune response that promotes the hardening of soft tissue (to wall off infectious agents) and the softening of hard tissue (to dissolve a substrate for growth of infectious agents). In our study, serum LDL cholesterol emerged as an independent factor that is associated significantly with femoral BMD, after adjusting for age and CA IMT.

Although several studies have shown that increasing BMD is related to a decreasing prevalence of echogenic plaque [36,37], our results found no significant differences between the four groups according to the plaque echogenicity in lumbar and femoral BMD.

There are several potential limitations to this cross-sectional study. First, the subjects were not recruited from the community at large, but were selected from patients who underwent bone density determinations. This selection bias may explain the relatively high prevalence of osteoporosis in the subjects studied. In fact, we have already shown that 31 to 39% of post-menopausal women had osteoporosis [38-40]. Another bias is related to the classification of plaques. Despite the fact that the reproducibility of plaque echogenicity was good, some misclassification may have occurred. Lastly, we have no direct histopathological demonstration that increased IMT is due to atherosclerosis. The arterial thickening might have been due to another, non-atherosclerotic arteriopathy. However, IMT measurements are still useful in that IMT is strongly correlated with the presence of coronary artery diseases.

## Conclusion

In brief, our results showed that decreased BMD is correlated with increased CA IMT in postmenopausal women, independently of confounding factors. We suggest that bone status should be evaluated in patients with vascular disease to assess whether preventive or therapeutic intervention is necessary.

## Competing interests

The authors declare that they have no competing interests.

## Authors' contributions

FA and NHH conceived the study and supervised its design, execution, and analysis and participated in the drafting and critical review of the manuscript. IH, FA and RA did data management and statistical analyses. All other authors enrolled patients, participated in data acquisition and critical revision of the manuscript. IH wrote the paper with input from all investigators. All authors read and approved the final manuscript.

## Pre-publication history

The pre-publication history for this paper can be accessed here:


